# Off-Pump Lung Transplantation: Key Surgical and Anesthetic Considerations

**DOI:** 10.1016/j.atssr.2025.07.019

**Published:** 2025-08-19

**Authors:** Yoshiya Toyoda, Akshay Chauhan, Gordon H. Morewood, Hiromu Kehara, Sean M. Baskin, Sriram Vijayapuri, Mikiko Senzai, Roh Yanagida, Kewal Krishan

**Affiliations:** 1 Department of Cardiovascular Surgery, Temple University, Philadelphia, Pennsylvania; 2 Department of Anesthesia, Temple University, Philadelphia, Pennsylvania

## Abstract

This article presents an off-pump lung transplantation technique via bilateral anterior thoracotomy. It details anesthesia management, tailored ventilation, and surgical steps to maintain hemodynamic stability. Avoiding extracorporeal support, this approach minimizes bleeding and enhances recovery, offering a safe, efficient alternative to clamshell incision for experienced transplant teams.

While lung transplantation is typically performed with extracorporeal support, select cases can be managed off-pump. This less-common approach offers potential benefits but requires close surgical-anesthetic coordination. We present our technique for off-pump bilateral lung transplantation via anterior thoracotomies.

## Technique

The patient’s cardiopulmonary data are reviewed. If pulmonary vascular resistance >5 Wood units or right ventricle (RV) dysfunction is present, epinephrine is given preinduction. Cerebral oximetry guides fluid and inotrope use during induction. After induction and placement of a double-lumen endotracheal tube, ventilation is disease-specific: Obstructive disease requires prolonged expiratory times to prevent breath stacking and cardiac compression (avoiding extrinsic positive end expiratory pressure), while fibrotic disease requires pressure control, shorter expiratory times, and high positive end expiratory pressure. Inhaled nitric oxide is started at induction. A peripheral arterial line, preferably brachial, is placed preinduction to avoid radial signal dampening during positioning. A dual-lumen internal jugular introducer is inserted postinduction for volume access and oximetric pulmonary artery (PA) catheter placement. Transesophageal echocardiography is used unless contraindicated.

The ventilation/perfusion scan helps determine the side of the lung to explant first in an off-pump lung transplant. The lung contributing less to overall gas exchange and perfusion is removed first. This minimizes hemodynamic instability and ensures that the better-perfused lung can sustain oxygenation while the new lung is being implanted. In cases with equal lung function on ventilation/perfusion scan, we implant the left lung first after dissecting the right hilum. This enables prompt intervention for postreperfusion instability, with easier access to central extracorporeal membrane oxygenation and right PA clamping if needed.

**Figure 1:**
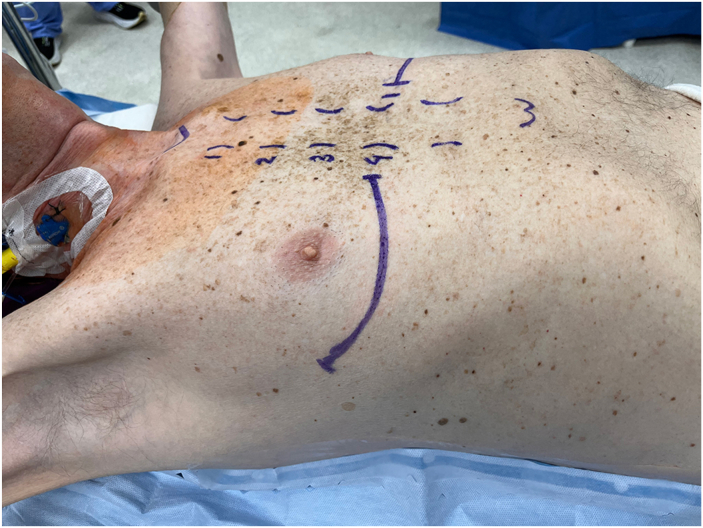
The patient is positioned supine with a scapular towel roll to extend the spine and widen intercostal spaces. Arms are abducted to expose axillary regions. Skin markings define the anterolateral thoracotomy, usually at the fourth intercostal space for pulmonary fibrosis and fifth for chronic obstructive pulmonary disease.

The patient is positioned as in Figure 1, and an ∼8 cm anteroaxillary incision is made along the fourth or fifth intercostal space, based on pathology and thoracic size. The ipsilateral lung is temporarily deflated to reduce injury risk. An Allison retractor or plastic-tip suction protects the lung while the intercostal muscle is divided to expand the rib space without fracturing ribs. In restrictive disease, a no. 2 vicryl (Ethicon) diaphragmatic traction suture is placed on the dome and passed through the skin to retract it posterolaterally, increasing space and maneuverability. A similar suture on the left pericardium (anterior to the phrenic nerve) retracts the heart medially to improve left hilar exposure.

**Figure 2:**
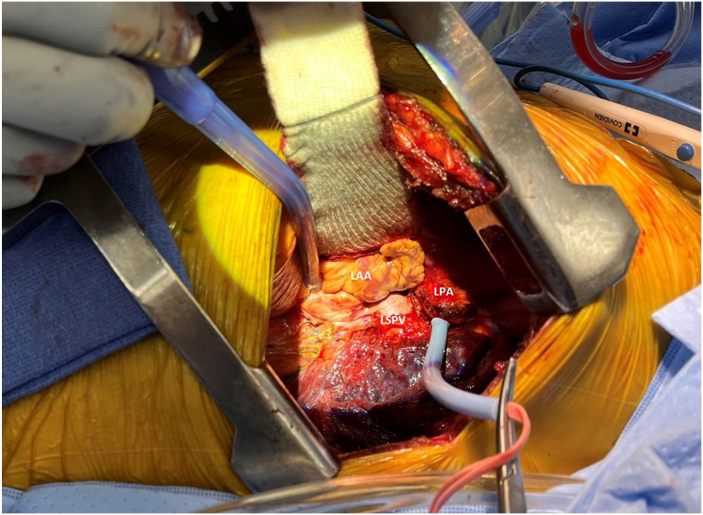
Dissected left lung hilum with snare around the left pulmonary artery for the pulmonary artery occlusion test. (LAA, left atrial appendage; LPA, left pulmonary artery; LSPV, left superior pulmonary vein.)

The hilum is carefully dissected, with the phrenic nerve identified to prevent injury. The inferior pulmonary ligament is clipped and divided. A plane is developed between the PA and superior pulmonary vein (PV), both looped with umbilical tapes. The pericardium is incised to loop the inferior PV. These steps are repeated contralaterally, ideally completed before donor lung arrival to minimize cold ischemia time. The PA is test-snared to assess hemodynamic stability; extracorporeal support is initiated if instability or RV failure occurs (Figure 2). The PA and PV are divided distally using Endo GIA staplers (Medtronic) to preserve length for clamping and anastomosis. Pneumonectomy is completed by sharply dividing the main bronchus proximal to the upper lobe origin. The PV stump is prepared by opening the pericardium circumferentially around the left atrium (LA). On the right, the PA is mobilized from the superior vena cava and azygos vein; on the left, it is mobilized from the aortic arch. The bronchial stump is refashioned to avoid excessive length and preserve vascularity, minimizing dissection and electrocautery.

Initial dissection and single-lung ventilation may cause notable hypercarbia and/or hypoxia, depending on disease severity. Acute respiratory acidosis is typically well tolerated, and intermittent arterial blood gas monitoring suffices. Patients with chronic end-stage hypoxia may tolerate lower oxygen saturations; with adequate hemoglobin, saturations as low as 70% can be acceptable if mixed venous oxygen saturations and RV function are closely monitored via transesophageal echocardiography. Hilar dissection may compress cardiac chambers, necessitating vigilant monitoring of cardiac output and perfusion pressure. Support with volume loading, vasopressors (vasopressin or norepinephrine), or inotropes (epinephrine) should be guided by PA and transesophageal echocardiography data. Close communication with the surgical team is critical during PA test occlusion to assess mixed venous oxygen saturations and RV function.

**Figure 3:**
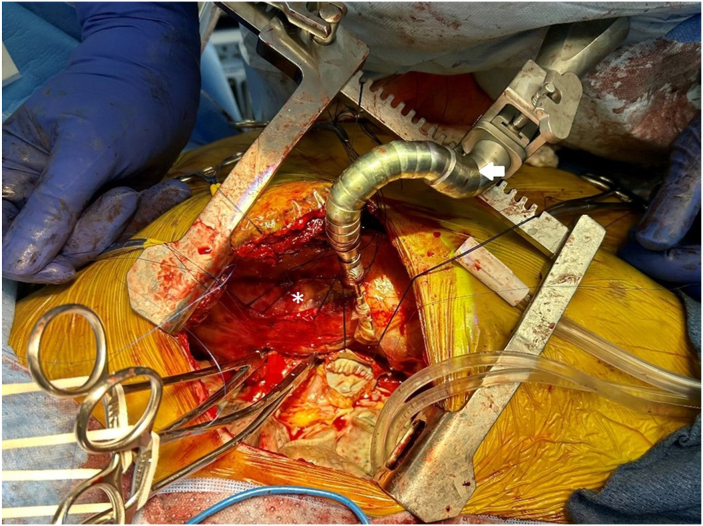
A Herc Flex (Terumo Cardiovascular) stabilizer arm (arrow) retracts mediastinal tissues during anastomosis, improving visualization while reducing cardiac pressure to maintain stability in off-pump lung transplantation. The pericardium is opened to expose the ascending aorta and right atrium (∗), potential sites for extracorporeal membrane oxygenation cannulation.

A cooling jacket is placed around the donor lung, which is positioned in the thoracic cavity. A Herc Flex (Terumo Cardiovascular) stabilizer arm retracts mediastinal tissues (Figure 3). Bronchial anastomosis is performed end-to-end with a running 4-0 PDS suture (Ethicon), reinforced by interrupted sutures at the membranous-cartilaginous junction. In size mismatch, donor bronchus intussusception into the recipient is preferred. Pericardium and connective tissue are approximated to cover and seal the anastomosis, preventing adhesions with PA and facilitating dissection in redo lung transplant. The PA is clamped with a Satinsky clamp, staple lines excised, and both PAs trimmed to prevent kinking. A running 5-0 Prolene (Ethicon) end-to-end anastomosis is performed without torsion, sutures left untied for de-airing. The recipient LA is clamped with a larger Satinsky clamp, requiring heart retraction and careful hemodynamic monitoring due to potential hypotension. Pulmonary venous orifices are aligned, and a cuff-to-cuff LA anastomosis is done with a running 4-0 Prolene suture, also left untied for de-airing. Methylprednisolone (500 mg) is given to reduce ischemia-reperfusion injury.

Before reperfusion, the patient's hemodynamic state must be optimized. Reperfusion causes intravascular volume loss, washout of metabolic byproducts, and microbubbles entering the systemic circulation. Hypovolemia should be corrected, and inotropes started if RV dysfunction is present. Post-reperfusion RV function is closely monitored; early epinephrine boluses may help manage transient dysfunction. The patient is placed in Trendelenburg position to reduce air embolism risk. The pulmonary artery clamp is partially released for gradual reperfusion and deairing through the untied LA suture line. The LA clamp is removed, maintaining a blood/saline interface to prevent air entry. After deairing, the LA suture line is secured, and the PA anastomosis sutures are tied. The PA clamp is gradually released over 5 minutes. Postreperfusion, a vasodilatory state may occur with elevated cardiac output and low systemic vascular resistance. Moderate to high doses of vasopressin and/or norepinephrine may be required, but this typically resolves quickly in the intensive care unit.

Initial lung inflation postreperfusion is done via a 10-15 second recruitment at 30 cm H_2_O. Lung expansion is confirmed visually. Ventilation is then started with positive end expiratory pressure 10, fraction of inspired oxygen 40%, respiratory rate 20, and tidal volume 6 mL/kg. If postreperfusion pulmonary edema severely compromises the first graft function, and a tidal volume under 300 mL or fraction of inspired oxygen above 60% is required to maintain peripheral oxygen saturation over 90%, then initiating venovenous extracorporeal membrane oxygenation may be preferable to avoid further injury to the new lung. Thoracotomies are closed after ensuring hemostasis and drain placement. Before leaving the operating room, the patient's volume status is optimized, and acid/base and electrolyte imbalances corrected. The double-lumen endotracheal tube is exchanged for a single-lumen tube and bronchoscopy is performed to clear airways and confirm bronchial anastomosis integrity.

## Comment

Lung transplantation without extracorporeal support is a safe, effective option for experienced teams, potentially reducing bleeding and operative times. A study comparing elective venoarterial extracorporeal membrane oxygenation to off-pump technique found no significant differences in perioperative transfusion, primary graft dysfunction, or length of stay^1^

Although the clamshell incision is common for its exposure and ease of aortic cannulation, we prefer bilateral thoracotomy for its advantages. It avoids sternum-related complications like infection and dehiscence,^2^ minimizes bleeding by preventing anterior mediastinal dissection, and improves postoperative pain and respiratory dynamics thanks to a smaller incision.^3^ This technique also shortens operative time by eliminating sternum stabilization^4^ and allows implantation of slightly oversized lungs without wound closure issues.
